# Entropic effect of macromolecular crowding enhances binding between nucleosome clutches in heterochromatin, but not in euchromatin

**DOI:** 10.1038/s41598-018-23753-0

**Published:** 2018-04-03

**Authors:** Inrok Oh, Saehyun Choi, YounJoon Jung, Jun Soo Kim

**Affiliations:** 1 Department of Chemistry, Seoul National University, Seoul, 08826 Republic of Korea; 2 Department of Chemistry and Nanoscience, Ewha Womans University, Seoul, 03760 Republic of Korea

## Abstract

Sharp increase in macromolecular crowding induces abnormal chromatin compaction in the cell nucleus, suggesting its non-negligible impact on chromatin structure and function. However, the details of the crowding-induced chromatin compaction remain poorly understood. In this work, we present a computer simulation study on the entropic effect of macromolecular crowding on the interaction between chromatin structural units called nucleosome clutches. Nucleosome clutches were modeled by a chain of nucleosomes collapsed by harmonic restraints implicitly mimicking the nucleosome association mediated by histone tails and linker histones. The nucleosome density of the clutches was set close to either that of high-density heterochromatin or that of low-density euchromatin. The effective interactions between these nucleosome clutches were calculated in various crowding conditions, and it was found that the increase in the degree of macromolecular crowding induced attractive interaction between two clutches with high nucleosome density. Interestingly, the increased degree of macromolecular crowding did not induce any attraction between two clutches with low nucleosome density. Our results suggest that the entropic effect of macromolecular crowding can enhance binding between nucleosome clutches in heterochromatin, but not in euchromatin, as a result of the difference in nucleosome packing degrees.

## Introduction

Cellular environments are highly crowded with biological macromolecules, such as proteins and RNAs, and it was shown that the change in the degree of macromolecular crowding could significantly influence the structural and dynamic properties of diverse biological processes^1–5^. Macromolecular crowding also has non-negligible impact on chromatin structure^5–11^, as suggested by experimental observations that sharp increase in macromolecular crowding induces a significant degree of chromatin compaction in the cell nucleus^12^. However, structural and mechanistic investigation of the crowding effect on chromatin has been hampered by the limited resolution of microscopic observations. It is presumed that the chromatin compaction accompanying the sharp increase in macromolecular crowding occurs due to entropy-driven effective attraction (also termed depletion effect) induced between structural units of chromatins by the presence of highly concentrated crowders. Detailed understanding of how chromatins respond to the change in macromolecular crowding may provide valuable information on the microscopic interactions involved in dynamic genome organizations.

Recently, computational studies confirmed that the crowding-induced effective attraction could significantly reduce the size of chromatin domains in more crowded environments^13,14^. In these studies, chromatins were modeled as chains of spherical units of several tens of nanometer diameter, representing the local assemblies of DNA and proteins. In the study on eukaryotic chromatins^13^, the use of such coarse-grained chains of spherical units of 30 nm diameter was justified by eukaryotic chromatin structures with rigid, well-ordered fiber-like nucleosome aggregates of 30 nm thickness, widely known as the 30-nm chromatin fiber^15–17^. However, the concept of the 30-nm chromatin fiber has been challenged in recent years due to the lack of *in vivo* experimental evidence^18–20^. Instead, it was suggested that chromatin is composed of irregularly folded 10-nm fibers of nucleosomes^20^. In addition, a recent experimental work using super-resolution nanoscopy reported the observation of dispersed clutches of nucleosomes, rather than the highly compacted, 30-nm fiber of nucleosomes^21^. If one considers the chromatin unit as irregularly folded, variable clutches of nucleosomes, the packing density of the irregularly folded nucleosome clutches may need careful consideration in the study of macromolecular crowding. Recently, we showed that the effective interaction between two collapsed chains in a crowded environment is significantly influenced by the packing density of the collapsed chains^22^, and the same is expected between the nucleosome clutches. Therefore, it is very timely to revisit the crowding effect on chromatin compaction by considering more structural details of nucleosome assemblies beyond the assumption of the 30-nm chromatin fiber.

In this work, we investigated crowding-induced effective interactions between irregularly folded nucleosome clutches by performing coarse-grained simulations. Nucleosome clutches^21^ were modeled by chains of nucleosomes collapsed by harmonic restraints, implicitly mimicking the nucleosome aggregation mediated by histone tails^23^ and linker histones^24^, in contrast to earlier computational studies^13^ where nucleosome aggregates of 30 nm scale were modeled by single spherical units. However, the structures of nucleosome clutches^21^ have yet to be determined, possibly with variable size and nucleosome density. We thus prepared a generic model of nucleosome clutches with variable nucleosome densities but with a fixed size. Typically, chromatin domains in the interphase eukaryotic nucleus are categorized into euchromatin and heterochromatin, based on the physical compaction state, gene density, and transcriptional activity^25,26^. Euchromatin is a less condensed, gene-rich, and transcriptionally active domain, whereas heterochromatin is condensed, gene-poor, and transcriptionally silenced. Therefore, the models of nucleosome clutches were prepared to have two nucleosome densities, one comparable to that of euchromatin, and the other close to that of heterochromatin. In addition, our model of nucleosome clutches was constructed with an emphasis on the excluded volume interactions of nucleosomes and crowders and the effect of macromolecular crowding presented in this work is solely based on the configurational entropy of nucleosomes and crowders. Our simulation results using the two models of nucleosome clutches revealed that the macromolecular crowding can have distinguishable entropic influences on structural units of euchromatin and heterochromatin, which could not have been appreciated without the models of detailed nucleosome assemblies. Our finding that the entropic effect of macromolecular crowding may enhance binding between chromatin units in heterochromatin, but not in euchromatin, may have significant impact on our understanding of genome organization.

The rest of this paper is organized as follows. In the following section, we describe the models of nucleosome clutches in euchromatin and heterochromatin and the simulation methods. Simulation results are presented in Section Results, where we first discuss the crowding-induced effective interactions and then discuss the decomposition of effective interactions to understand the distinct effect of crowding on euchromatin and heterochromatin clutches. This work is summarized in Section Discussion.

## Methods

### Models of nucleosome clutches

In general, it is known that lengthy DNA molecules in cell nucleus are compacted in a hierarchical manner: primarily, by binding and wrapping histone protein complexes to form an array of nucleosomes (also called a 10-nm fiber of nucleosomes); secondarily, by clustering intra-chromatin nucleosomes to form clutches or irregular aggregates of nucleosomes; further, by folding and looping to compartmentalize into domains; and finally, by forming chromosome territories. Here, we assumed that the large-scale chromatin compaction induced by macromolecular crowding arises from bottom-up aggregation of smaller structural units such as clutches or irregular aggregates of nucleosomes at a few tens of nanometer scale. The effect of macromolecular crowding on the large-scale chromatin compaction is then inferred from the crowding effect on the effective interaction between the nucleosome clutches.

**Figure 1:**
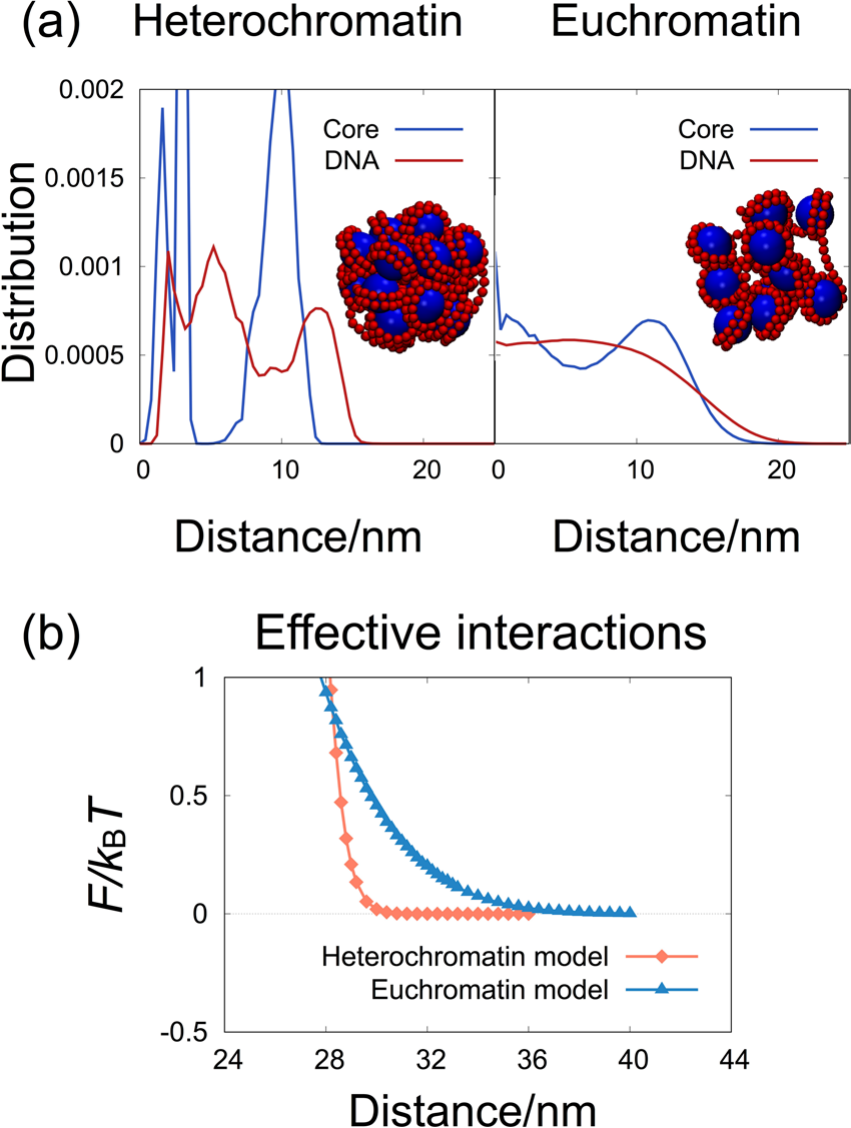
(**a**) Snapshots of each nucleosome clutch model, and the distributions of nucleosome core particles and DNA monomers from the center of mass in the absence of crowders. The snapshots are taken by VMD^49^. (**b**) The effective interaction, *F*, between a pair of nucleosome clutches as a function of pair distance in the absence of crowders.

The nucleosome clutches were modeled as a collapsed chain of nucleosomes. A chain of nucleosomes was modeled by a complex of a single chain of double-stranded DNA and spherical nucleosome core particles, as shown in snapshots of Fig. 1(a). The DNA was modeled as a linear chain of DNA monomers with a diameter of 2 nm, corresponding to 6 basepairs (bp). Each nucleosome core particle represents an octameric complex of histone proteins, and is of 7 nm diameter. All non-bonded pairs of the particles interact with each other through repulsive, excluded volume interaction, which was modeled by a repulsive part of the Lennard-Jones (LJ) potential, as shown below.

1 $$U(r)=\{\begin{array}{ll}4\varepsilon [{(\frac{\sigma }{r-{r}_{0}})}^{12}-{(\frac{\sigma }{r-{r}_{0}})}^{6}]+{\varepsilon } & 0 < r-{r}_{0} < {r}_{c}\\ 0 & {\rm{elsewhere}}\end{array}$$

where *σ* is a unit of length equal to 2 nm, *r*_0_ determines the size of particles, and *r*_*c*_ is the cut-off length of the LJ interaction. To employ the pure repulsion in the LJ potential, the value of *r*_*c*_ was taken to be 2^1/6^*σ*, at which the LJ potential reaches the minimum. The values of *r*_0_ for a pair of DNA monomers and for a pair of nucleosome core particles were set as 0 and 2.5*σ*, respectively, so that their diameters are approximately defined as 1*σ* and 3.5*σ*. The value of *r*_0_ for a pair of a DNA monomer and a nucleosome core particle was 1.25*σ*. The crowders were modeled to have the same diameter of 7 nm as the nucleosome core particles. DNA monomers are bonded through a combination of the finite extension nonlinear elastic (FENE) potential, shown in Eqn. (2), and the repulsive LJ potential shown in Eqn. (1).

2 $${U}_{{\rm{FENE}}}(r)=-\frac{1}{2}{k}_{{\rm{FENE}}}{R}_{b}^{2}\,\mathrm{ln}(1-{(\frac{r}{{R}_{b}})}^{2})$$

where *k*_FENE_ is 30 *k*_B_*T*/*σ*^2^ and *R*_*b*_ is 1.5*σ*, chosen to prevent the mutual crossing of monomer-monomer bonds in the DNA chain. In addition, the two consecutive bonds are subject to the harmonic angle potential as ${U}_{{\rm{angle}}}=\frac{1}{2}{k}_{{\rm{angle}}}{({\theta }-{{\theta }}_{0})}^{2}$, where *θ* is an angle between two consecutive bond vectors, and *θ*_0_ is 0, to ensure the stiffness of DNA. *k*_angle_ is chosen as 25 *k*_B_*T*/*rad*^2^ to mimic the persistence length of 50 nm.

A chain of nucleosomes was modeled by binding nucleosome core particles regularly on a single DNA chain. Each nucleosome core particle is wrapped about 1.75 turns by 24 DNA monomers (144 bps) through strong harmonic restraints (with harmonic constants of 1000 *k*_*B*_*T*/*σ*^2^) between a nucleosome core particle and DNA monomers. Harmonic restraints are also applied (with the same harmonic constants) between neighboring DNA monomers to keep two loops of DNA monomers around the nucleosome core particle close and in parallel. Such nucleosome units are separated by five linker DNA monomers (30 bps). This model was recently used in the study of chromatin structure confined in an array of nanoposts^27^. Although more detailed models have been proposed to study the chromatin structure^28–31^, our model is efficient to investigate the entropic effect of volume occupation either by nanostructure confinement^27^ or by crowders.

Nucleosome clutches of euchromatin and heterochromatin domains were then modeled by collapsed chains of 10–20 nucleosomes, as shown in snapshots of Fig. 1(a). Each chain of nucleosomes was collapsed by using additional harmonic restraints to pull all nucleosome cores toward the center-of-mass of the nucleosome chain, such that the diameter of the spherically-collapsed nucleosome chain becomes close to 30 nm. The application of the harmonic restraints to collapse the nucleosome chain is phenomenological rather than biological, coarsely mimicking the nucleosome chain folding through the inter-nucleosome association mediated by histone tails^23^ and linker histones^24^. Due to the application of the harmonic restraints, the oligomeric nucleosome chains were collapsed to spherical, distinct entities without connection to other nucleosomal arrays in the chromatin. It was assumed that the effective interactions calculated between collapsed nucleosome clutches mimic those between the nucleosome clutches that are not adjacent along the chromatin but located close due to chromatin looping. In addition, it was previously shown that the crowding-induced effective interaction between a pair of spherical units in different oligomeric chains are almost identical to those between a pair of independent, spherical objects^32^ and, thus, our results obtained for a pair of distinct nucleosome clutches can be used to understand the crowding effect on the nucleosome clutches in chromatins in spite of the ignorance of the connection to other nucleosomal arrays in our model.

The collapsed chain diameter of 30 nm was chosen to compare the effects of macromolecular crowding on the chromatin structural units composed of 10–20 nucleosomes at the nucleosome densities found in euchromatin and heterochromatin, but not to model the 30-nm chromatin fiber that is being replaced by the concept of irregularly folded 10-nm fiber in recent years^20^. We also confirmed that the change of the collapsed chain size for both nucleosome densities of euchromatin and heterochromatin domains did not alter our qualitative conclusions on the entropic effect on the pair interactions, by performing extra simulations of larger collapsed chain models with diameter of 45 nm (data not shown). Although the size of nucleosome clutches can also be variable in euchromatin and heterochromatin, our focus in this work is the influence of the packing density of collapsed nucleosome clutches. Therefore, it is fair to compare the crowding effect in euchromatin and heterochromatin by varying the packing density of nucleosome clutches while keeping the size the same, especially, in the absence of detailed information on the size of nucleosome clutches in euchromatin and heterochromatin. In this work, the models of nucleosome clutches in euchromatin and heterochromatin contain 11 and 20 nucleosomes, respectively, such that the density of nucleosomes compacted in a spherical volume of 30 nm diameter is close to the overall nucleosome densities estimated for euchromatin and heterochromatin domains. The nucleosome densities of heterochromatin and euchromatin domains were reported, assuming the 30 nm-chromatin fiber, to be 11 nucleosomes per 11 nm fiber length and 6 nucleosomes per 11 nm fiber length, respectively^33^.

Figure 1(a) shows snapshots of both nucleosome clutches and the radial distributions of nucleosome cores and DNA monomers in each nucleosome clutch in the absence of crowders. The heterochromatin clutch is so tightly compacted that nucleosome cores have well separated radial distributions. The euchromatin clutch is compacted more loosely, so that the distributions are relatively smooth. The distributions of both models vanish roughly at 15 nm, suggesting that the diameters of the nucleosome clutches are close to 30 nm.

Figure 1(b) shows the effective interaction between a pair of nucleosome clutches, *F*, in the absence of crowders. *F* was measured as a function of separation *ξ* as shown in Eqn. (3).

3 $$F(\xi )=F(\infty )-{k}_{{\rm{B}}}T\,\mathrm{ln}\,\langle \exp \,[-{U}_{{\rm{inter}}}(\xi )/{k}_{{\rm{B}}}T]\rangle ,$$

where *U*_inter_(*ξ*) represents the interaction energy calculated for a pair of nucleosome clutches placed at a distance of *ξ*^32,34^. Conformations of a single nucleosome clutch were generated from the MD simulations, and a pair of the configurations were chosen and placed at a distance of *ξ* to calculate the value of *U*_inter_(*ξ*). We generated at least 10^6^ pair configurations to measure an ensemble-averaged 〈exp[−*U*_inter_(*ξ*)/*k*_B_*T*]〉 at each distance. Since only steric repulsions were considered between all units of the nucleosome clutches, the effective interaction is purely repulsive at short pair distances. The repulsive interaction can also be interpreted as the entropic effect of nucleosome clutches. Our model of a nucleosome clutch is a chain of nucleosomes collapsed by the harmonic restraint around its center-of-mass, as shown in Fig. 1(a), and a large number of chain conformations are available. When a pair of the nucleosome clutches come very close, many overlapping conformations are prohibited, which reduces the number of available chain conformations. The reduced number of conformations implies the decrease in conformational entropy of nucleosome clutches, resulting in pure repulsion between a pair of nucleosome clutches at short distances.

The repulsive interaction is relatively sharp for heterochromatin clutch, and is softer for euchromatin clutch. The repulsive interaction vanishes near 29 nm for the heterochromatin clutch, whereas it decays out to 34 nm for the euchromatin clutch. This difference is attributed to the different packing degree of the clutch models: the heterochromatin model is tightly compacted with sharp boundary, whereas the euchromatin model is loosely compacted with irregular surface boundary. It is simply noted that the effective interactions shown in Fig. 1(b) are not fitted to either exponential or power-law functions, which unfortunately prevents the development of coarse-grained potential models with a simple functional form.

### Simulations

Calculations of effective interactions, termed the potential of mean force (PMF), were achieved separately for the cases with and without crowders. In the absence of crowders, the effective interactions *F* arising from the conformational entropy of nucleosome clutches was calculated as described above in Eqn. (3). In the presence of crowders, the total effective interaction *F*_T_ was calculated via the constraint-biased molecular dynamics (MD) simulation^32,35,36^. *F*_T_ was calculated at various crowder volume fractions *ϕ*_*c*_, as shown in Eqn. (4):

4 $${F}_{{\rm{T}}}(\xi )={F}_{{\rm{T}}}(\infty )+{\int }_{\infty }^{\xi }[{\langle {f}_{c}\rangle }_{s}+\frac{2{k}_{{\rm{B}}}T}{s}]ds,$$

where *ξ* is the distance between a pair of nucleosome clutches. At each pair distance of *s*, the constraint force *f*_*c*_ was calculated by the constraint-biased MD simulation performed at each *ϕ*_*c*_, and averaged to 〈*f*_*c*_〉_*s*_, where 〈…〉_*s*_ denotes the ensemble average over all nucleosome clutches and crowder configurations at a constant pair distance of *s*. More details on the PMF calculation using Eqn. (4) have been discussed in previous works^32,35,36^. The MD simulations at each pair distance were performed at least 10^8^ MD steps with a time step of *dt* = 0.005*τ*_*MD*_ to obtain an ensemble-averaged constraint force. Here, *τ*_*MD*_ is a unit of time for the MD simulations, and is defined by $\sigma \sqrt{m/\varepsilon }$, where *σ*, *m*, and *ε* are unit length, mass, and energy. All MD simulations were performed at constant *N*, *V*, and *T* conditions by GROMACS version 5.1.2^37^. The nose-hoover thermostat was employed to maintain 1*k*_B_*T* of the temperature. The cubic simulation box with each dimension of 90 nm was larger than twice the maximum distance of the *F*_T_ measurement.

The total effective interaction *F*_T_ was then decomposed into two entropic contributions, one from nucleosome clutches and the other from crowders, as discussed in the section Results. An entropic contribution of nucleosome clutches *F*_NUC_ was calculated in the same way as used to calculate the effective interactions *F* in the absence of crowders but using the conformations of nucleosome clutches sampled in the presence of crowders. Eqn. (3) was used to obtain *F*_NUC_ from 10^6^ configurations of a pair of nucleosome clutches. By subtracting *F*_NUC_ from *F*_T_, a pure entropic contribution of crowders *F*_CRD_ was obtained^22^.

## Results

### Crowding-induced effective interactions

**Figure 2:**
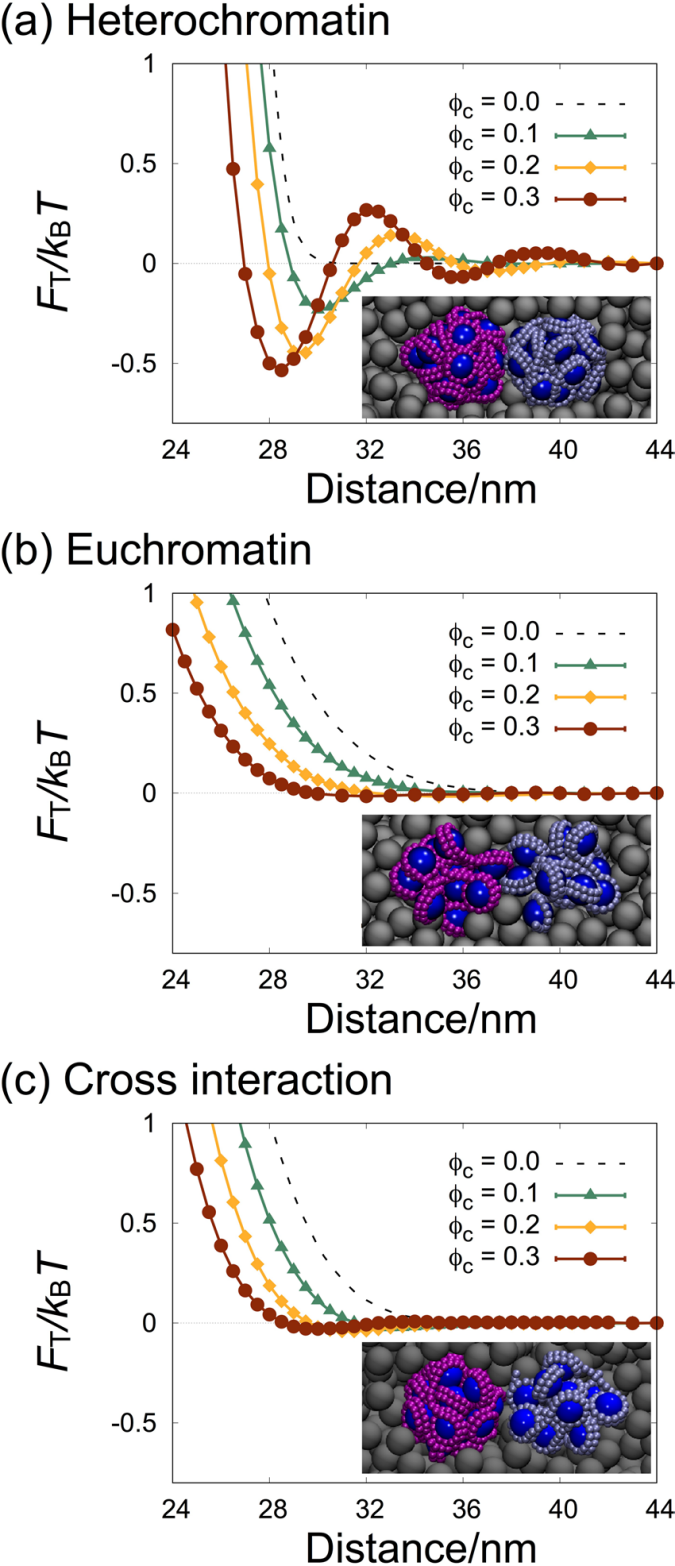
The potential of mean forces (PMF) between a pair of nucleosome clutches at various crowder volume fractions *ϕ*_*c*_ between 0.0 and 0.3, (**a**) between a pair of heterochromatin clutches, (**b**) between a pair of euchromatin clutches, and (**c**) between a mixed pair of heterochromatin and euchromatin clutches. Representative snapshots are taken by VMD^49^ and shown in inset. Error bars are smaller than the symbols.

In this work, we investigated the crowding effect on the pair interaction between nucleosome clutches as a model of local interaction sites in chromatins, with the aim of understanding the effect of macromolecular crowding on the large-scale chromatin compaction. The effective interaction (PMF) between a pair of nucleosome clutches was calculated by performing the constraint-biased MD simulations for a pair of nucleosome clutches in the presence of highly concentrated crowders, as shown in Fig. 2. The crowders were considered as spherical particles of 7 nm diameter, and the simulations were performed at varying crowder concentrations in terms of the volume fraction of crowders *ϕ*_*c*_ between 0.0 and 0.3. The size of crowders was chosen to be the same as that of nucleosome core particles, close to that of a spherical particle with a molecular weight of 70 kDa (the average of nucleoplasmic proteins) and with a partial specific volume of 0.73 ml/g.

It is noted that our models do not consider the electrostatic interactions arising from electric charges of histone cores, histone tails, DNA, crowders, and salt ions in aqueous solutions, but focus on the excluded volume interactions of nucleosomes and crowders. In addition, the roles of histone tails and linker histones in inter-nucleosome associations are, as mentioned above, implicitly mimicked by the application of harmonic restraints that collapse chains of nucleosomes into a spherical volume to match nucleosome densities in euchromatin and heterochromatin. Therefore, our simulation results in this work are solely based on the configurational entropy of nucleosomes and crowders which depend on the excluded volume interactions of nucleosomes and crowders at different concentrations of crowders and at different nucleosome densities in nucleosome clutches.

Figure 2(a) and (b) show the PMFs, represented by *F*_T_, for pairs of heterochromatin clutches and euchromatin clutches at varying crowder volume fractions, respectively, and Fig. 2(c) is the data of the PMFs for a mixed pair of heterochromatin and euchromatin clutches. The effective potentials in the absence of crowders shown in Fig. 1(b) are also presented for comparison (dashed lines in Fig. 2).

The PMFs for the pair of heterochromatin clutches in Fig. 2(a) show the change in effective interaction by the presence of highly concentrated crowders. As *ϕ*_*c*_ increases from 0.0 through 0.3, three noticeable changes are observed. First, the repulsive barrier shifts towards shorter separation, implying that due to the presence of crowders, the size of nucleosome clutches is reduced, and the clutches are more compacted. Second, the effective attractions represented by negative values are developed just beyond the repulsive core. This becomes more significant with increase in *ϕ*_*c*_. Finally, small repulsive bumps with positive values are developed at slightly larger separations, which increases with *ϕ*_*c*_.

Among these changes, the development of the effective attraction at higher *ϕ*_*c*_ is most interesting, because the effective attraction induced by the presence of high concentrations of crowders has been known to play important roles in the phase separation and aggregation in colloid-polymer mixtures. Consistent with earlier studies on crowding or the depletion effect on colloid phase behavior, the effective attractions in Fig. 2(a) increase with *ϕ*_*c*_. The crowding effect can be qualitatively interpreted, relying on the theory by Asakura and Oosawa (AO)^5,38,39^. The AO theory estimates the effective attraction between two hard sphere colloids in crowded solutions, based on the entropy gain of repulsive crowders upon the association of two hard-core, spherical colloids. Around each colloid in a solution of crowders, there occurs a spherical volume (whose radius is the sum of the colloid and crowder radii) that is inaccessible to crowders, called the excluded volume. The association of two colloids results in the overlap of their excluded volumes, and thus allows slightly larger volume available for crowders, leading to the increase in the configurational entropy of crowders. The AO theory predicts that the strength of effective attraction is proportional to *ϕ*_*c*_. Our simulation results show that the attraction strength increases with *ϕ*_*c*_, in qualitative agreement with the AO theory. However, the increase is not directly proportional to *ϕ*_*c*_, but becomes diminished as *ϕ*_*c*_ increases from 0.1 through 0.2 to 0.3, not in quantitative agreement with the theory.

The PMFs calculated for the pair of euchromatin clutches in Fig. 2(b) show more dramatic deviation from the predictions of the AO theory. The effective potential never goes negative at all *ϕ*_*c*_’s, which suggests that the macromolecular crowding does not induce any effective attraction between euchromatin clutches. This is in sharp contrast to the AO prediction that the entropy-driven effective attraction becomes proportionally stronger with crowder volume fraction. As *ϕ*_*c*_ increases, the only change in the effective potential is the shift of the repulsive barrier towards shorter distance, which is ascribed to the compaction of each clutch due to volume occupation by crowders. The effect of macromolecular crowding is the same for a mixed pair of euchromatin and heterochromatin clutches, as shown in Fig. 2(c), without any effective attraction between the mixed pair.

Our results were obtained by the simulations of a pair of isolated nucleosome clutches, whereas actual nucleosome clutches in chromatins are not isolated but connected to one another along chromatins. Our previous simulations on chromatin models with structural units of 30 nm diameter showed that the entropy-induced crowding effect on isolated structural units is not significantly different from those on the connected units in chains^32^. Therefore, our results obtained for a pair of isolated nucleosome clutches can then be used to understand the crowding effect on chains of nucleosome clutches.

In addition, we assumed that the nucleosome clutches are distinct without any inter-clutch nucleosome-nucleosome association. This assumption can be acceptable when one focuses on the work required to bring a separated pair of nucleosome clutches close to each other (which is the definition of PMF). It provides information of how stable a pair of nucleosome clutches can become due to macromolecular crowding when they are brought close. Our results showed that the effect of macromolecular crowding induces effective attraction between heterochromatin clutches whereas pure repulsion between euchromatin clutches. In reality, however, when nucleosome clutches are brought close, there may occur any inter-clutch nucleosome-nucleosome association, helping the formation of larger-scale chromatin structures. Our results based on the distinct nucleosome clutches suggest that the crowding-induced attraction between heterochromatin clutches helps keep the nucleosomes in different clutches close to each other for a prolonged period of time so that they have a better chance for associative interaction between the inter-clutch nucleosomes. On the other hand, macromolecular crowding induces pure repulsion between euchromatin clutches and therefore does not help the association of the nucleosomes in different clutches. These lead us to conclude that the entropic effect of macromolecular crowding enhances binding between nucleosome clutches in heterochromatin, but not in euchromatin.

These results on the heterochromatin and euchromatin clutches may have a significant impact on our understanding of dynamic genome organization. Previous experimental studies on the crowding-induced chromatin compaction could not distinguish the structural changes in different domains of chromatins. Although not yet clear, the distinguishable crowding effects on heterochromatin and euchromatin may have unappreciated biological implications.

### Decomposition of effective interactions

The physics behind the distinguishable effects of macromolecular crowding on heterochromatin and euchromatin clutches can be explained by separating the total effective interaction into entropic contributions from nucleosome clutches and crowders. The entropic contribution of nucleosome clutches arises from the overlapping of clutch conformations, and thus the reduced number of available conformations when they come close to each other, resulting in the repulsive interaction at short distances. On the other hand, the configurational entropy of crowders increases upon association of nucleosome clutches in crowded environments, leading to the effective attraction at short distances.

**Figure 3:**
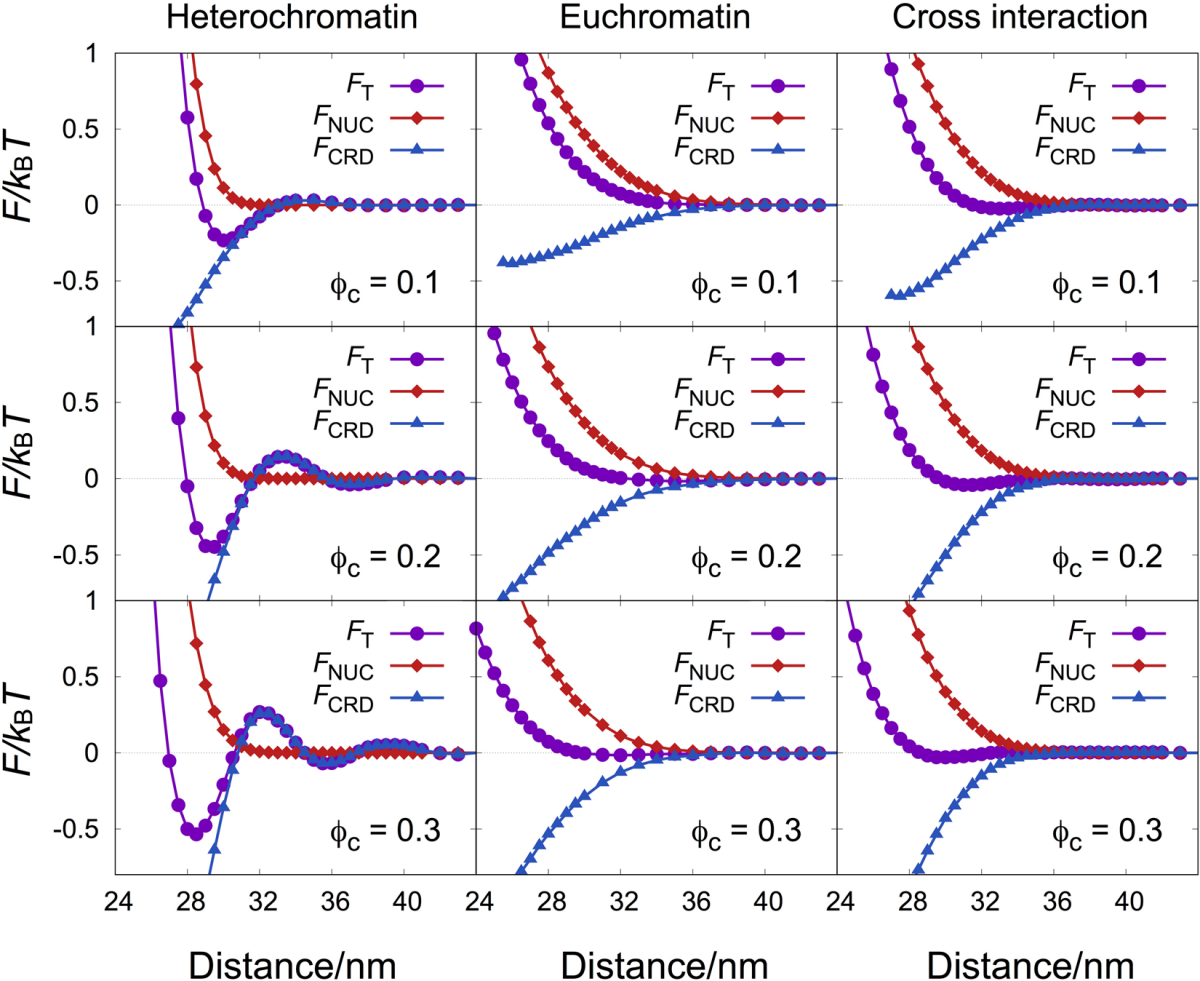
The total effective interaction (*F*_T_) is decomposed into two components of repulsion (*F*_NUC_) and attraction (*F*_CRD_), which are contributed from entropy changes in nucleosome clutches and crowders, respectively. The data in the left column are for a pair of heterochromatin clutches, those in the middle are for an euchromatin pair, and those in the right column are for a mixed pair of heterochromatin and euchromatin clutches at *ϕ*_*c*_ = 0.1, 0.2, and 0.3. *F*_NUC_ and *F*_CRD_ are presented as diamonds and triangles, respectively, whereas *F*_T_ is depicted as circles.

For separation of the two entropic contributions, we calculated the purely repulsive interaction from the entropic effect of nucleosome clutches, *F*_NUC_, and subtracted it from the total effective interaction, *F*_T_, so that the effective attraction from the entropic effect of crowders, *F*_CRD_, can be separately obtained. *F*_NUC_ was calculated in the same way as used for the calculation of *F* in the absence of crowders, as shown earlier in Eqn. (3), in which the change in the number of allowed clutch conformations was counted at each clutch-clutch distance and interpreted as the free energy change at the distance. In this case, however, all clutch conformations were obtained from the MD simulations of a single nucleosome clutch in the presence of crowders. *F*_CRD_ was then obtained by subtracting *F*_NUC_ from *F*_T_. Figure 3 shows the separate contributions to the total effective interaction, together with the total effective interaction.

In all three cases, at short distances, the entropic contribution from nucleosome clutches, *F*_NUC_, is purely repulsive, while that from crowders, *F*_CRD_, is attractive. Each repulsive and attractive interaction changes monotonically as *ϕ*_*c*_ increases. The repulsion at very close distance shifts slightly to shorter distance, implying the slight compaction of nucleosome clutches due to macromolecular crowding (which is also consistent with the slight changes in the distributions of nucleosome core particles and DNA monomers with varying degrees of macromolecular crowding, as shown in Figure S1 of the Supplementary Information). The effective attraction becomes stronger proportionally to *ϕ*_*c*_, consistent with the prediction from the AO theory quantitatively as well as qualitatively.

Comparing each contribution of the three models at the same *ϕ*_*c*_, two differences are easily recognized. First, the effective repulsion, *F*_NUC_, and attraction, *F*_CRD_, change more smoothly for the euchromatin pair and the mixed pair than for the heterochromatin pair. This can be attributed to the loose nucleosome packing and irregular surface in the euchromatin clutch model. On the other hand, the effective repulsion and attraction for the tightly packed heterochromatin clutch change very sharply, similar to those known for hard sphere colloids.

The second difference between the heterochromatin and euchromatin results is the cancellation of the repulsive and attractive interactions. For the heterochromatin model, the total effective interaction *F*_T_ obtained by adding *F*_NUC_ and *F*_CRD_ looks after each contribution at different regions, because the repulsion and attraction do not overlap much. In particular, the effective attraction at intermediate distances is clearly shown in *F*_T_. However, for the euchromatin model, *F*_T_ is purely repulsive, without showing any attractive feature induced by the entropic contribution of crowders. This is due to the overlap of the repulsion and attraction canceling out most of the attractive interaction. The overlap and cancellation of repulsion and attraction originate again from the loose nucleosome packing and consequent conformational flexibility in the euchromatin clutch model, as has been explained for loosely collapsed, generic polymer chains in our previous work^22^. As a result of the conformational fluctuation, the entropy-induced repulsion and attraction averaged over all possible configurations of nucleosome clutches and crowders have a broader interaction range leading to the significant overlap, unlike a pair of hard spherical objects with clearly separated repulsion and attraction.

Unlike our finding that softer euchromatin clutches have no effective pair attraction while more tight heterochromatin clutches have non-negligible crowding-induced attraction, Shendruk and coworkers^40^ reported that softer spherical particles have more enhanced crowding-induced attraction than the spherical particles with a steeper potential. Our euchromatin clutch model has the variable shape and size so that the crowding-induced attraction can occur at shorter distances^22^, which leads to complete overlapping and cancellation between *F*_NUC_ and *F*_CRD_ and, thus, the purely repulsive effective interaction *F*_T_.On the other hand, the particles in the work by Shendruk and coworkers^40^ have a spherically symmetric, repulsive potential that defines the fixed shape and size of the particle. The crowding-induced attraction occurs only outside the particle boundary and, as a result, the overlapping and cancellation of the attractive *F*_CRD_ with the repulsive *F*_NUC_ is limited and the total *F*_T_ shows the attractive well as well as the repulsive wall.

## Discussion

Understanding how genes are spatially organized in cell nucleus and orchestrated for specific biological functions remains a major challenge^26,41,42^. Chromatin organization is largely mediated by binding of scaffold proteins, such as SMC^43^, CTCF^44^, and HP1 proteins^45^. However, the importance of the non-specific, entropy-driven crowding effect has recently also been appreciated in elucidating the genome organization^5–11^. Experimentally, the effect of macromolecular crowding on chromatin organization has been investigated by means of cell incubation in media with different osmolarity. When incubated in the hypertonic medium, the cell shrinks due to the osmotic loss of water to the medium and a significant degree of chromatin compaction was observed^12,46,47^. Since protein transport across the cell membrane during the osmosis is limited, the reduction in cell volume at fixed amounts of proteins and RNAs was interpreted as an increase in the degree of macromolecular crowding. Therefore, the abnormal chromatin compaction was attributed to the large increase in the degree of macromolecular crowding^12^.

A change in cell volume is ubiquitous in a variety of regulatory processes, including regulation of metabolism, hormone release, cell proliferation, and cell death, and can lead to significant change in macromolecular crowding^48^. Understanding the abnormal chromatin compaction observed in crowded conditions can provide a new opportunity to understand the cell volume regulatory mechanism mediated by the change in chromatin organization. However, the understanding from the experimental studies of the osmotic-induced chromatin compaction has unfortunately been limited by the resolution of microscopic observations on the scale of a few hundred nanometers. No detailed structural information has been available that can distinguish different responses of chromatin domains, such as euchromatin and heterochromatin, to the change in macromolecular crowding.

Using the coarse-grained models of nucleosome clutches, we have shown, for the first time, that the response of different chromatin domains to the change in macromolecular crowding can be clearly distinguished. The model of heterochromatin clutches revealed that the elevated level of macromolecular crowding induces effective attraction between nucleosome clutches that may help binding in a crowded environment. On the other hand, the model of euchromatin clutches did not show any effective attraction between nucleosome clutches, which suggests that the euchromatin domain may not be subject to a significant structural change at elevated crowding levels. The apparent absence of effective attraction in the euchromatin clutch model was explained by the overlap of effective repulsion and attraction that arises from the soft nature of the euchromatin clutch model. On the other hand, the heterochromatin clutch model is dense with nucleosomes, so that the crowding effect induces an effective attraction similar to that induced between a pair of hard sphere colloids.

The aim of this work was to investigate the effect of macromolecular crowding on the pair interaction between a pair of chromatin structural units, called nucleosome clutches in this work, from which the crowding-induced, large-scale chromatin compaction is inferred. Our model was basically built on the excluded volume interactions between nucleosome clutches and crowders, while ignoring any molecular interactions, such as electrostatics. Therefore, the calculated effective interactions were induced strictly by the entropy change in nucleosome clutches and crowders, and did not include contributions from direct molecular interactions. The entropy-driven effective attraction calculated for the heterochromain clutch model was on the scale of roughly 0.5 *k*_B_*T* at *ϕ*_*c*_ = 0.20 and 0.30. This effective attraction may not be strong enough to induce any large-scale collapse-swelling transition of chromatins in higher order. However, the macromolecular crowding is not the sole modulator of chromatin organization, but complementary to direct molecular interactions between DNA and associated proteins^47^. Therefore, the small attraction of 0.5 *k*_B_*T* may become a significant addition to direct interactions, to induce large-scale changes in chromatin organization.

## Electronic supplementary material

Supplementary information
