# Supplementary material for: Entropic effect of macromolecular crowding enhances binding between nucleosome clutches in heterochromatin, but not in euchromatin
Source: Sci Rep. 2018 Apr 3;8:5469. doi: 10.1038/s41598-018-23753-0 (PMC5882907; doi:10.1038/s41598-018-23753-0)
Supplement: Supplementary file 1 — Supplementary information [file 41598_2018_23753_MOESM1_ESM.pdf]

Supplementary Information

"Entropic effect of macromolecular crowding enhances binding between nucleosome clutches in heterochromatin, but not in euchromatin"

Inrok Oh[1], Saehyun Choi[2], YounJoon Jung[1,*], Jun Soo Kim[2,+]

[1.] Department of Chemistry, Seoul National University, Seoul 08826, Republic of Korea

[2.] Department of Chemistry and Nanoscience, Ewha Womans University, Seoul 03760, Republic of Korea

Correspondence:

yjjung@snu.ac.kr

jkim@ewha.ac.kr

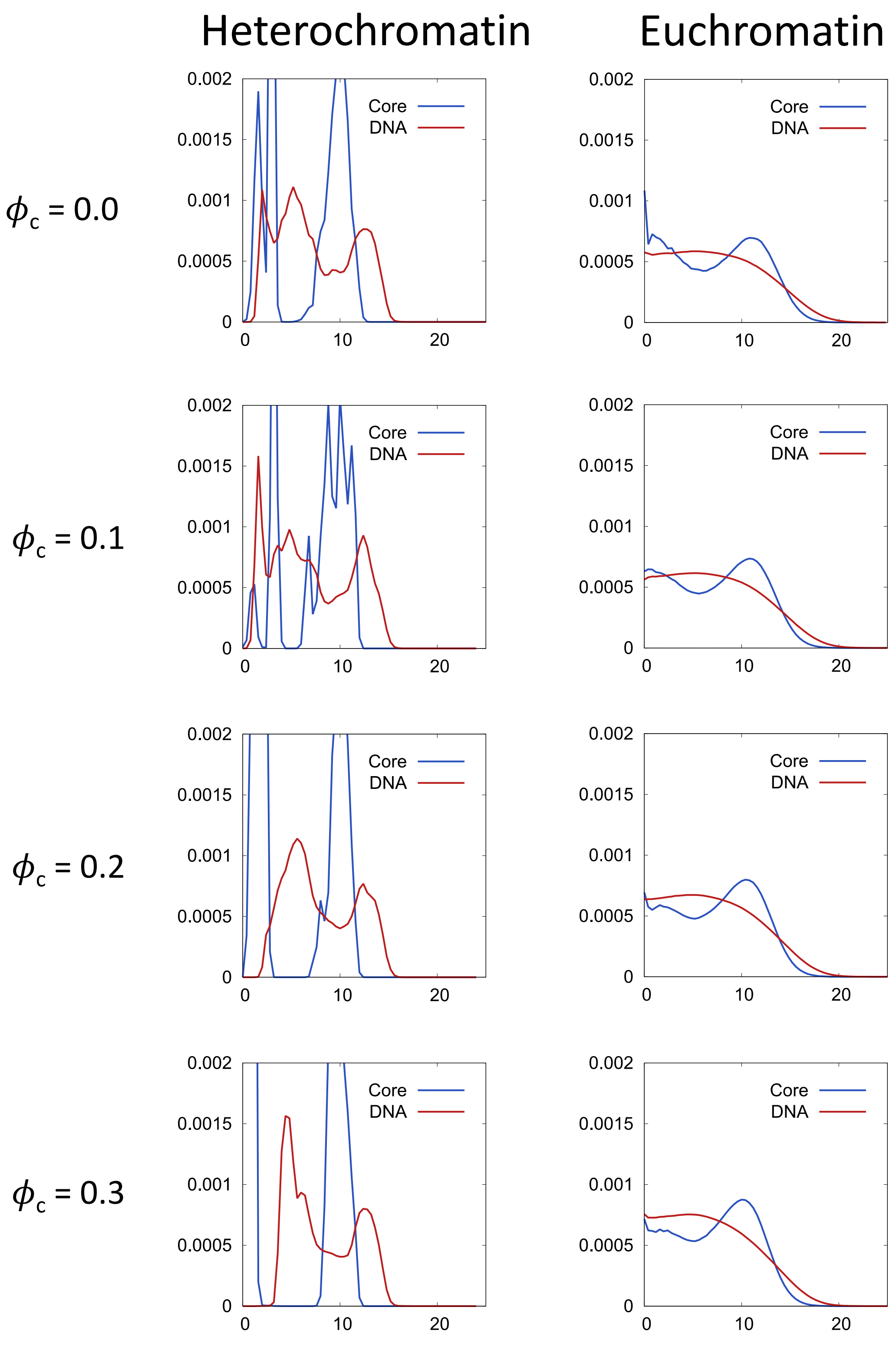

Figure S1. The distribution of nucleosome core particles and DNA monomers in models of nucleosome clutches at various crowding conditions of ϕ_c, sampled every 10^3 steps from the molecular dynamics simulations of a single nucleosome clutch in varying crowding conditions of ϕ_c, with simulation durations of 10^9steps.
